# Food hygiene practices and determinants among food handlers in Ethiopia: a systematic review and meta-analysis

**DOI:** 10.1186/s41182-022-00423-6

**Published:** 2022-05-19

**Authors:** Demisu Zenbaba, Biniyam Sahiledengle, Fikadu Nugusu, Girma Beressa, Fikreab Desta, Daniel Atlaw, Vijay Kumar Chattu

**Affiliations:** 1 Public Health Department Bale-Goba, Madda Walabu University Goba Referral Hospital, Bale Goba, Ethiopia; 2 School of Medicine, Anatomy Department, Madda Walabu University Goba Referral Hospital, Bale Goba, Ethiopia; 3 Centers for Trans Disciplinary Research, Saveetha Institute of Medical and Technical Sciences, Saveetha University, Chennai, 600077 India; 4 Department of Community Medicine, Faculty of Medicine, Datta Meghe Institute of Medical Sciences, Wardha, 442107 India

**Keywords:** Food safety, Food hygiene, Food handler, Good practice, Ethiopia

## Abstract

**Background:**

Food-borne diseases are a major public health concern worldwide, particularly in low and middle-income countries (LMICs), such as Ethiopia. Poor food hygiene practices primarily exacerbate food-borne illness transmission. Prior studies on the food hygiene practices among food handlers in Ethiopia were inconsistent. Therefore, this meta-analysis and systematic review aimed to estimate the pooled proportion of good food hygiene practices and identify the determinants in Ethiopia.

**Methods:**

The preferred reporting items for systematic review and meta-analysis (PRISMA) instruments were used, and a systematic search was performed in the PubMed/MEDLINE, POPLINE, HINARI, Science Direct, Cochrane Library databases, and Google Scholar were systematically last searched on the 24th February 2022 for relevant articles. Only the observational studies that reported the proportion of good food hygiene practices and their associated factors among food handlers were included. The quality of the included studies was assessed by two independent authors. Articles with unclear methodologies and did not report the overall proportions of good food hygiene practice were excluded. The effect estimates for pooled proportion and pooled odds ratio (POR) along with a 95% confidence interval (CI) were determined conducting using DerSimonian–Laird's random effect model.

**Results:**

Among 817 retrieved studies, 23 eligible articles with a total sample size of 7153 study participants were included in the meta-analysis. The pooled proportion of good food hygiene practices among food handlers was 50.5% [95% CI: (41.6, 59.4%]; *I*^2^ = 98.7%, *p* value = 0.001]. Food handlers with formal education (POR = 4.60, 95% CI: 3.05, 6.93), good knowledge (POR = 1.98, 95% CI: 1.26, 3.11), training (POR = 3.52, 95% CI: 2.35, 5.28), and a positive attitude (POR = 3.41, 95% CI: 2.52, 4.61) about food hygiene components, as well as regular medical checkups (POR = 6.75, 95% CI: 4.49) were significantly associated with good food hygiene practice.

**Conclusions:**

Only half of Ethiopia's food handlers had good food hygiene practice.

**Implication of the study:**

The key elements of effective food hygiene practice that will aid in the development of feasible interventions to increase food handler compliance with food hygiene components have been identified.

**Supplementary Information:**

The online version contains supplementary material available at 10.1186/s41182-022-00423-6.

## Background

Food availability and safety for all people at all levels are necessary for developing a productive workforce that leads to a nation's rapid economic, social, and sustainable growth [1, 2]. Food hygiene refers to “a collection of fundamental concepts used to maintain environmental conditions during the storage, processing, and preparation of food” [3]. One of the most common causes of foodborne disease outbreaks, ranging from diarrhea to cancer is mainly due to improper food handling and hygiene standards [4, 5].

Foodborne illnesses are a major public health concern in both developed and developing countries. Diarrheal diseases, mostly caused by microbial infections found in food or water, continue to be the major cause of illness and death globally [5, 6]. The eating of contaminated food is responsible for 70% of diarrheal illness. According to the World Health Organization, food borne diseases affect up to 30% of the population in rich nations each year, while up to 2 million people die in low- and middle-income countries (LMICs) [5, 7, 8]. More than 200,000 people die from intestinal parasite infections in Africa, exacerbated by poor sanitation and hygiene standards [9–14]. In Ethiopia, the prevalence of food-borne infections among food handlers ranges from 14.5 to 44.1% [15–18]. These food-borne infections are responsible for a significant increase in economic expenditures and strain on countries' healthcare systems [19].

Although disease transmission by food handlers is a prevalent and chronic concern worldwide, they also serve a critical role in guaranteeing food safety [20]. In locations, where personal hygiene and environmental sanitation are lacking, parasitic diseases remain a serious public health concern [10, 21, 22]. Food handlers with poor personal hygiene who work in food establishments can easily become infected with enteric pathogens, and their hands, in particular, might serve as a vector for the spread of dangerous microorganisms during or after gastrointestinal infection [23–25]. In this regard, Food handlers' lack of proper food handling standards is blamed for about 75% of food-borne illness outbreaks, according to available evidence [26–28].

Ensuring food safety to protect public health remains a top priority in developed and developing countries [2]. Poor food hygiene practices primarily exacerbate food-borne illness transmission [29–32]. Previous studies showed inconsistent good food hygiene practices among food handlers in Ethiopia, ranging from 19.4 to 90.4% [33–36]. On the other hand, knowledge, attitude, training on main food hygiene components, and routine medical checkups of food handlers were some of the factors associated with good food hygiene practices [34, 37, 38]. This systematic review and meta-analysis aimed to estimate the pooled proportion of good food hygiene practices and associated factors among food handlers working in food and drinking establishments in Ethiopia.

## Methods

### Registration and protocol

The Preferred Reporting Items for Systematic Reviews and Meta-Analysis (PRISMA) checklist was used to conduct this systematic review and meta-analysis (Additional file 1). The study protocol was registered in PROSPERO (Record ID: CRD42021287598).

### Search strategy

A comprehensive search of databases was undertaken using PubMed/MEDLINE, POPLINE, HINARI, Science Direct, Cochrane Library databases, and Google Scholar from publication year of 8th March 2012 to 30th October 2021 to find potentially relevant articles. All searches were limited to papers written in English and last search in all databases were performed on the 24^th^ February 2022. In addition to the electronic database search, grey literature was searched using Google search, and the Addis Ababa University Digital Library. We also searched the reference lists of the included articles for related studies. For the PubMed/MEDLINE search, the following phrases and keywords were used: [“Food OR Foods AND Hygiene OR “Hand hygiene” AND “Professional Practices” OR Practice AND “Epidemiologic Factors” OR Factor OR Determinant OR Determinants, OR “Epidemiologic Determinants” OR “Factors, Epidemiologic” AND “Food Handling” OR “Food handlers” AND Ethiopia] as well as all possible combinations of these terms. We used database-specific subject headings linked with the above terms and keywords used in PubMed for the other electronic databases.

### Eligibility criteria

#### Inclusion and exclusion criteria

Articles that met the following criteria were considered for inclusion in the review. The study included at least two and above food hygiene components, such as personal hygiene habits, such as hand washing at critical times, fingernail clipping, wearing protective clothing, utensil cleaning and sanitizing, and waste management practices.

##### Language

Only papers written in the English language were taken into consideration.

##### Study setting

Studies conducted in Ethiopia.

##### Study population

The study involved all food handlers working in food establishments, including institutions, such as universities and prisons.

##### Study design

All observational studies (cross-sectional, case–control, and cohort) that reported the proportion of good food hygiene practices and associated factors were considered.

##### Publication status

Both published and unpublished studies were included.

#### Exclusion criteria

Articles with unclear methodologies, studies conducted among housewives in the community, full-text papers not fully available after at least two personal email contacts with the corresponding authors, and articles that did not indicate the overall proportion of good food hygiene practice were all excluded.

### Outcome variables assessment

There are two main outcomes in this study: the primary outcome variable was good food hygiene practice, which was characterized as having a good practice based on the operational definition of included studies. The total number of food handlers who had good food hygiene practices was divided by the total number of food handlers participating in the study and multiplied by 100, which was used to calculate the proportion of good food hygiene practices. The second objective of this review was to determine the determinants of good food hygiene practice. Accordingly, the following factors food handlers' Educational status (formal and no formal education), knowledge (good and poor), attitude (positive and negative), training (yes and no), and routine medical checkup were examined.

### Study selection and data extraction

All the articles for this review were imported into EndNote version X8, and duplicates were removed. Data extraction was performed using the JBI data extraction format [39, 40]. Based on the predefined inclusion criteria, two authors (DZ and BS) independently assessed and identified papers by their titles, abstracts, and full texts. The screened items were then compiled, and any differences were handled through consensus. The data extraction format included the primary author, publication year, region, study area, sampling method, data collection method, cut off point to categorize food hygiene practice, major food hygiene components assessed by primary studies, sample size, response rate, and proportion of good food hygiene practice. For the second outcome, data were extracted into a two-by-two table.

### Quality assessment

The Joanna Briggs Institute (JBI) meta-analysis of statistics assessment and review instrument (MAStARI) quality evaluation tool was used to assess the quality of the appended studies [40]. The JBI parameters include an appropriate sampling frame, proper sampling technique, study subject and setting description, sufficient data analysis, use of valid methods for the identified conditions, a valid measurement for all participants, using appropriate statistical analysis, in a valid and reliable outcome measure, with a 50% or higher overall score considered low risk of bias. Accordingly, risks of bias were categorized as low (total score of ≤ 2), moderate (total score of 3–4), or high (total score of > 5) in terms of their likelihood [40]. The quality of the included studies was assessed by two independent authors (DZ and BS). Any discrepancy that arose was resolved by consensus. Finally, papers with a score of 5 or higher, indicating a high risk of bias, were ruled out (Additional file 2). The grade of studies reported significant determinants of good food hygiene practice was performed using relative effect (OR) in which quality status ranged from low to moderate (Additional file 3).

### Data synthesis strategy

The data were extracted into a Microsoft Excel file before being analyzed. STATA software, version 16, was used for data analysis. The standard errors of the included studies were calculated using the following formula $\left( {{\text{SE}}\, = \,\surd p\left( {{1}\, - \,p} \right)/n} \right)$. The *I*^2^ statistics and the *p* values of the Cochrane *Q* test were used to explore heterogeneity in the reported proportion. The *p* values of the Cochrane *Q* test < 0.1 deemed the presence of heterogeneity among studies. We have applied the Higgins *I*^2^ test statistics to calculate the percentage of total variance due to heterogeneity across studies [40]. Although there is no exact criterion for when heterogeneity becomes significant, some researchers recommend low heterogeneity when I^2^ values are between (25–50%), moderate (50–75%), and high (> 75%) [40]. The DerSimonian-Laird's impact was evaluated using a random-effects model, because the test statistic revealed substantial heterogeneity among the studies (*I*^2^ = 98.7%, *p* value = 0.001). The effect sizes were expressed as proportion and odds ratio along with 95% confidence interval (CI). After calculating standard error, the natural logarithm (ln) adjusted odds ratio and 95% confidence levels of each included article were used to determine the association between good food hygiene practice and its determinants. According to the indicated category of *I*^2^, there was a huge variety between the studies included in this review. We conducted subgroup analysis by region, study area, sampling method, sample size, data collection technique, and cut off points to categorize food hygiene practice to identify the possible source of heterogeneity. The forest plot was used to display the meta-analysis results. A funnel plot was used in conjunction with meta-regression to investigate publication bias. In the absence of publication bias, the plot resembles an asymmetrical, large, inverted funnel. To objectively examine publication bias, Egger's weighted regression and Begg's rank correlation tests (*p* value < 0.05) were applied, but neither of them was found to be statistically significant. A leave-one-out sensitivity meta-analysis was performed to assess the robustness of the findings.

## Results

**Fig. 1:**
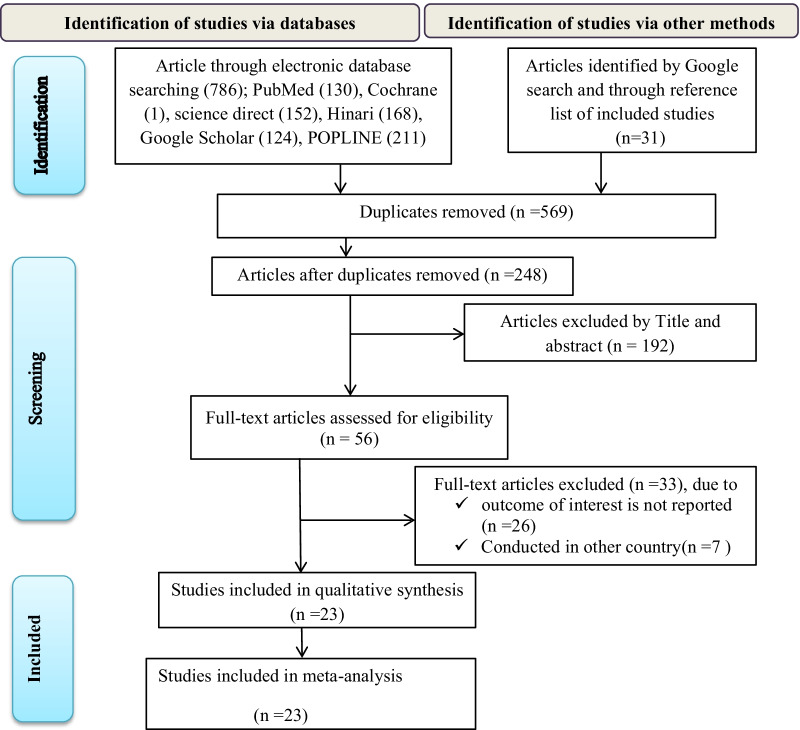
Flow chart of study selection for systematic review and meta-analysis of food hygiene practice among food handlers in Ethiopia

The systematic literature search resulted in the retrieval of 817 articles. Of these, 569 duplicates were removed, and 248 articles were evaluated based on title and abstract. After excluding 186 articles, a total of 56 full-text articles were screened for eligibility based on the pre-set criteria, and 33 articles were excluded. Finally, 23 eligible articles were included in the meta-analysis [33–38, 41–57] (Fig. 1).

### Description of the included studies

The included studies were cross-sectional by design and were published between 2012 and 2021. A total of 7153 study participants were included in the current meta-analysis to estimate the pooled proportion of good food hygiene practices among food handlers. Regarding the regional distribution of the included studies, twelve (12) studies were from the Amhara region [36–38, 42–44, 47, 51–55] and six from Oromia [33–35, 49, 56, 57], two from Addis Ababa [41, 50], two from the Tigray region [45, 48], One from the Southern Nations, Nationalities, and Peoples Region (SNNPR) [46], and one from Somali region [34] (Table 1).

**Table 1:** Descriptive summary of 23 studies included in the meta-analysis to estimate good food hygiene practice among food handlers in Ethiopia

Study ID	Author (year)	Region	Sampling method	Component of food hygiene assessed by each study	Response rate (%)	sample size	The proportion of good food hygiene practice with 95%
1	Abdi et al. 2021 [41]	Addis Ababa	Simple random sampling	hand, utensils and water hygiene	95.2	394	27.4 (26.7, 28.1)
2	Abe et al. 2021[56]	Oromia	Simple random sampling	hand, utensils and water hygiene	99	305	57.7 (52.2, 63.2)
3	Meleko et al. 2015 [50]	Addis Ababa	Census	Personal/Hand and Utensil hygiene	100	302	47.6 (46.7, 48.5
4	Adane et al. 2018 [42]	Amhara	Systematic sampling	Hand and utensils hygiene	100	135	69.6 (68.4, 70.7)
5	Azanaw et al. 2019 [38]	Amhara	Simple random sampling	waste management, Utensil cleanliness	100	384	49.0 (48.2, 49.8)
6	Chekol et al. 2019 [37]	Amhara	Simple random sampling	not reported	98.6	416	40.1 (39.4, 40.9)
7	Dagne et al. 2019 [43]	Amhara	Simple random sampling	Wearing protective cloth, clean and sanitize working service, finger nail trimming, utensil and hand hygiene	100	423	49.6 (48.8, 50.4)
8	Derso et al. 2017 [44]	Amhara	Simple random sampling	Personal, Hand and Utensil hygiene	98.8	417	67.6 (66.9, 68.3)
9	Gizaw et al. 2014 [53]	Amhara	Systematic sampling	Wearing protective cloth, utensil and hand hygiene	100	403	30.3 (29.6, 31.0)
10	Kibret et al. 2012 [36]	Amhara	Simple random sampling	Hand hygiene	100	455	90.1 (89.8, 90.4)
11	Lema et al. 2020 [47]	Amhara	Simple random sampling	Wearing protective cloth, utensil and hand hygiene	98.2	394	46.7 (45.9, 47.5)
12	Reta et al. 2018 [51]	Amhara	Simple random sampling	Wearing protective cloth, utensil hygiene and finger nail trimming	100	288	46.5 (45.6, 47.4)
13	Alemayehu et al. 2020 [54]	Amhara	Simple random sampling	Wearing protective cloth, utensil hygiene and finger nail trimming	100	408	53.7 (52.9, 54.5)
14	Teferi et al. 2021[57]	Oromia	Simple random sampling	not reported	100	422	50.5 (45.7, 55.3)
15	Tessema et al. 2020[55]	Amhara	Census	Wearing protective cloth, utensil and hand hygiene	94.4	406	52.5 (51.7, 53.3)
16	Yenealam et al. 2020 [52]	Amhara	Systematic sampling	Working environment cleaning, utensil and hand hygiene	95.53	214	66.4 (65.4, 67.4)
17	Kuti et al. 2015 [35]	Oromia	Census	Wearing protective cloth, utensil and hand hygiene, finger nail trimming,	98	198	90.4 (89.8, 91.0)
18	Yeshanew et al*.* 2021 [33]	Oromia	Simple random sampling	Wearing protective cloth, utensil and hand hygiene	100	139	19.4 (18.4, 20.4)
19	Mekasha et al. 2016 [49]	Oromia	Simple random sampling	working environment cleanliness, utensil and hand hygiene	100	112	41 (39.7, 42.3)
20	Lalit et al. 2015 [45]	Tigray	Simple random sampling	finger nail trimming, hand hygiene	97.5	369	53.1 (52.3, 53.9)
21	Mardu et al*.* 2020 [48]	Tigray	Census	Environmental and hand hygiene	100	66	51.5 (50.0, 53.0)
22	Legesse et al. 2017 [46]	SNNPR	Simple random sampling	wearing protective cloth, utensil and finger nail trimming, hand hygiene	98.9	383	32.6 (31.9, 33.3)
23	Tesfaye et al. 2020 [34]	Somali	Census	Personal and hand hygiene, finger nail trimming	100	120	27.5 (26.3, 28.7)

*SNNPR* Southern Nations, Nationalities, and peoples’ Region

### The proportion of good food hygiene practice

**Fig. 2:**
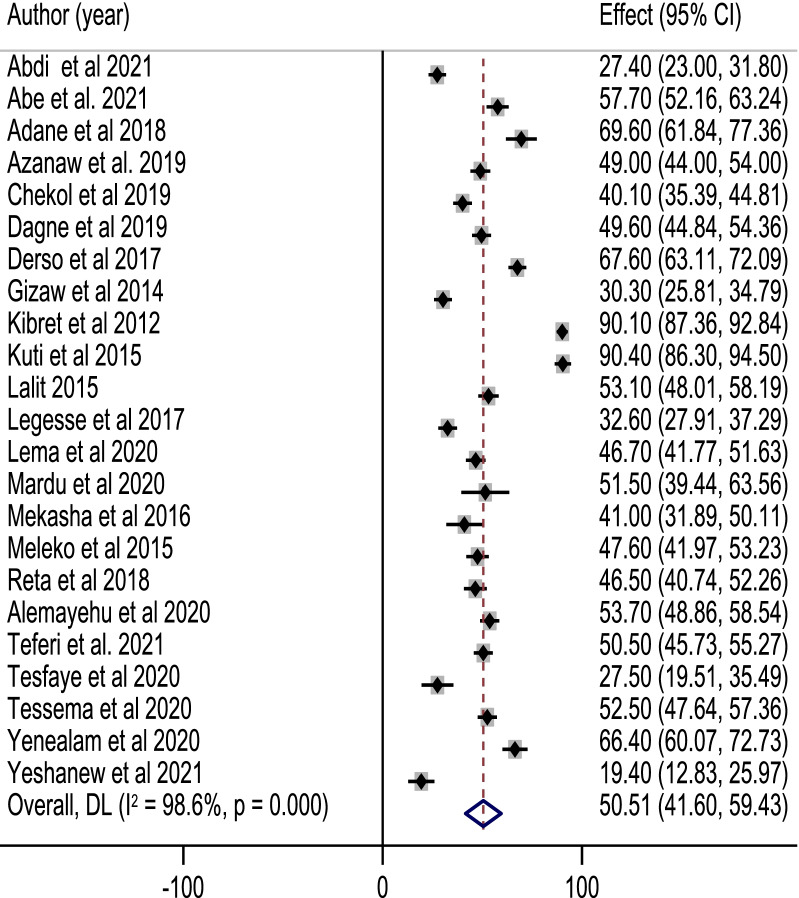
Forest plot of pooled prevalence of good food hygiene practice among food handlers in Ethiopia

In this meta-analysis, the pooled proportion of good food hygiene practices among food handlers in Ethiopia was 50.5%; 95% CI: (41.6, 59.4%). High heterogeneity was observed across the included studies (*I*^2^ = 98.7%, *p* < 0.001). As a result, a random effect model was used to estimate the pooled proportion of good food hygiene practices among food handlers in Ethiopia. The highest proportion of good food hygiene practice was 90.4%; (95% CI: 89.8, 91.0%) reported by Kuti et al., [35], whereas the lowest proportion of food hygiene practice was 19.40; (95% CI: (12.83, 25.97%) reported by Yeshanew et al. [33] (Fig. 2). A univariate meta-regression analysis was done utilizing variables, such as year of publication, quality score, and sample size to identify potential sources of heterogeneity. Of included variables, the year of publication was identified as a significant source of heterogeneity (Table 2).

**Table 2:** Factors with the heterogeneity of food hygiene practice among food handlers in the current meta-analysis based on univariate meta-regression

Variable	Coefficient	*p* value	95% CI
Year of publication	− 2.87	0.038	− 5.59, − 0.152
Sample size	0.01	0.749	− 0.05, 0.072
Response rate	− 0.667	0.772	− 5.19, 3.852
The quality score of the study	− 7.154	0.265	− 19.76, 5.437

### Sensitivity analysis

**Fig. 3:**
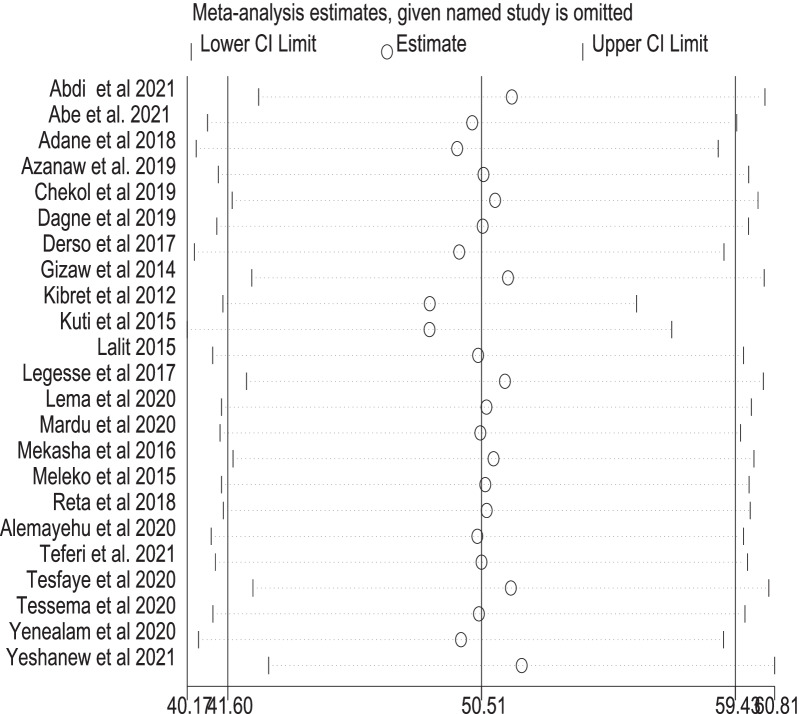
Sensitivity analysis of the level of food hygiene practice removed at a time: Prevalence and 95% confidence interval of good food hygiene practice among food handlers in Ethiopia

A leave-one-out sensitivity analysis was used to test the findings' reliability. The sensitivity analyses revealed that using the random-effects model was robust, and no single study affected the pooled proportion of good food hygiene practices among food handlers. After a single study was removed from a meta-analysis, the pooled proportion of good food hygiene practice was close to the actual effect size (Fig. 3).

### Publication bias

**Fig. 4:**
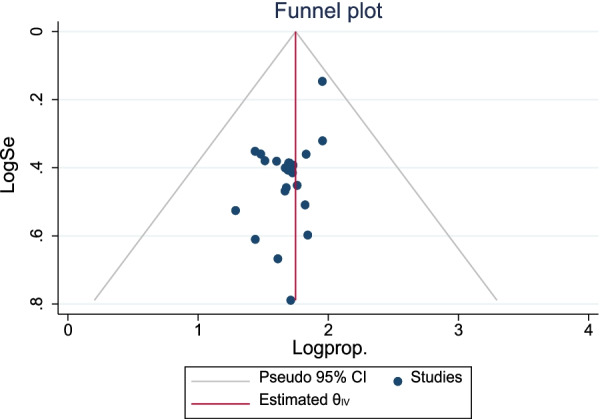
Funnel plot with 95% confidence limits of the pooled proportion of food hygiene practice among food handlers in Ethiopia

The publication bias was assessed using the funnel plot. The funnel plot revealed that the distribution of articles was uniform. We used Begg's and Egger's based tests objectively to corroborate the asymmetry. Egger's and Begg's tests revealed no evidence of publication bias in the proportion of good food hygiene practices among food handlers (Egger's test, *p* = 0.124 and Begg's test, *p* = 1.084) (Fig. 4).

### Subgroup analysis

This meta-analysis performed subgroup analysis based on the country's regions, study setting, and sample size. Accordingly, the highest proportion of good food hygiene practice was observed in the Amhara region with a proportion of 55.2% (95% CI: 43.4, 66.9), followed by the Tigray region, 52.9 (95% CI: 48.2, 57.6%). We also conducted a subgroup analysis based with the study setting. The pooled proportion of good food hygiene practice was 49.9% in studies conducted exclusively in towns, while it was 61.6% among studies carried out in universities. The proportion of good food hygiene practices was 51.8% (95% CI:( 36.9, 66.8)) and 49.5 (95% CI: (37.8, 62.2)) among studies having a sample size of < 311 and ≥ 311, respectively. The pooled proportions of good food hygiene practice among studies used cut off point, greater than or equal to mean value and 50% to report good food hygiene practice was 47.3 and 53.4%, respectively. Of all subgroup analyses, a significant source of heterogeneity was observed among regions, sampling methods and cut off points to categorize food hygiene practice of included studies (Table 3).

**Table 3:** Subgroup analysis regarding proportion of food hygiene practices among Ethiopia's food handlers (2012–2021)

Variables	Subgroup	No of included study	Sample size	Proportion Good food hygiene practice (95% CI)	Heterogeneity across the studies		Heterogeneity between group (*p* value)
					*I*^2^ (%)	*p* value	
Region	Amhara	12	4343	55.2 (43.4, 57.2)	98.7	< 0.001	< 0.001
	Addis Ababa	2	696	37.4 (17.6, 61.8)	96.7	< 0.001	
	Oromia	6	996	47.9 (27.2, 70.5)	98.9	< 0.001	
	Tigray	2	435	52.9 (48.2, 57.6)	0	0.811	
	SNNPR	1	383	32.6(27.9, 37.3)	0		
Study area	City	2	778	38.2 (17.0, 59.3)	97.5	< 0.001	0.611
	Town	17	5415	49.9 (39.8, 60.1)	98.6	< 0.001	
	University	3	894	61.6 (31.2, 67.4)	99.2	< 0.001	
	Prison	1	66	51.5 (39.4, 63.6)	0		
Sample size	< 311	10	1879	51.8 (36.9, 66.8)	98.1	< 0.001	0.809
	≥311	13	4852	49.5 (37.8, 62.2)	98.90	< 0.001	
Sampling method	Census	5	3059	54.0 (31.1, 77.0)	98.9	< 0.001	< 0.001
	Simple random sampling	15	5309	48.4 (37.5, 59.3)	98.7	< 0.001	
	Systematic sampling	3	752	55.3 (28.0, 72.7)	98.4	< 0.001	
Data collection method	Interview	10	2682	50.0 (36.6, 63.4)	98.3	< 0.001	0.806
	Interview and observation	10	3624	52.5 (37.4, 67.7)	99.0	< 0.001	
	Observation	3	847	45.5 (30.7, 60.4)	94.7	< 0.001	
Cutoff point used to categorize food hygiene practice	≥mean	6	2398	47.3 (41.0, 53.6	90.0	< 0.001	< 0.001
	≥ 50%	14	3942	53.4 (39.9, 66.8)	99.0	< 0.001	
	Not reported	3	813	43.5 (21.7, 65.3)	97.7	< 0.001	

*SNNPR* Southern Nations, Nationalities, and peoples’ Region

### Factors associated with food hygiene practice

**Fig. 5:**
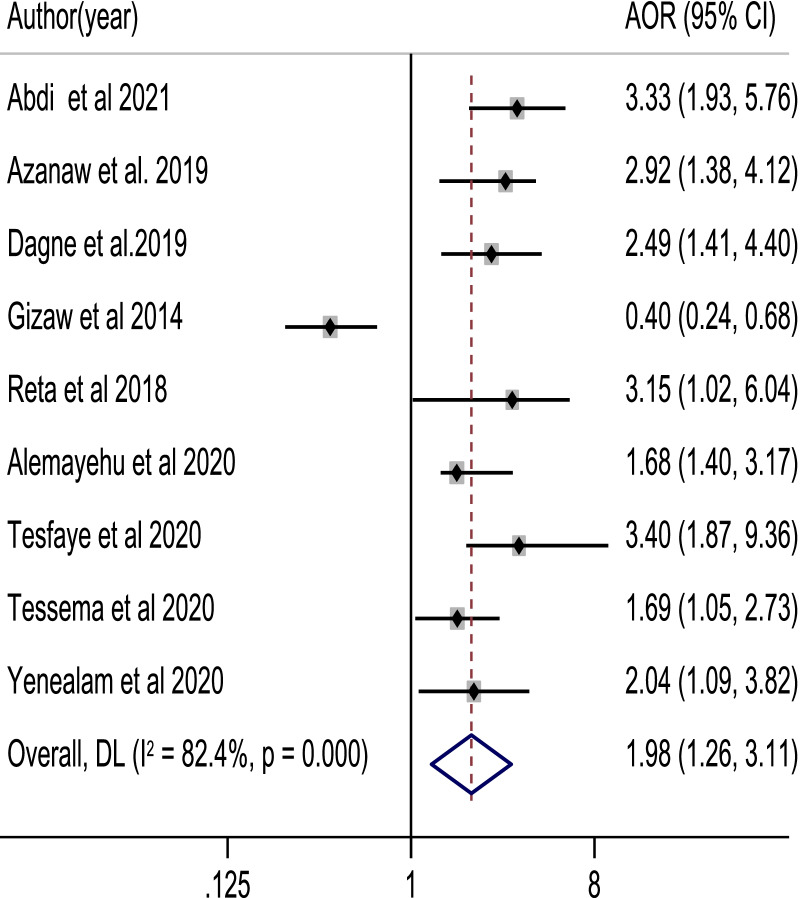
The pooled adjusted odds ratio of the association between good knowledge of food handlers and food hygiene practice in Ethiopia

**Fig. 6:**
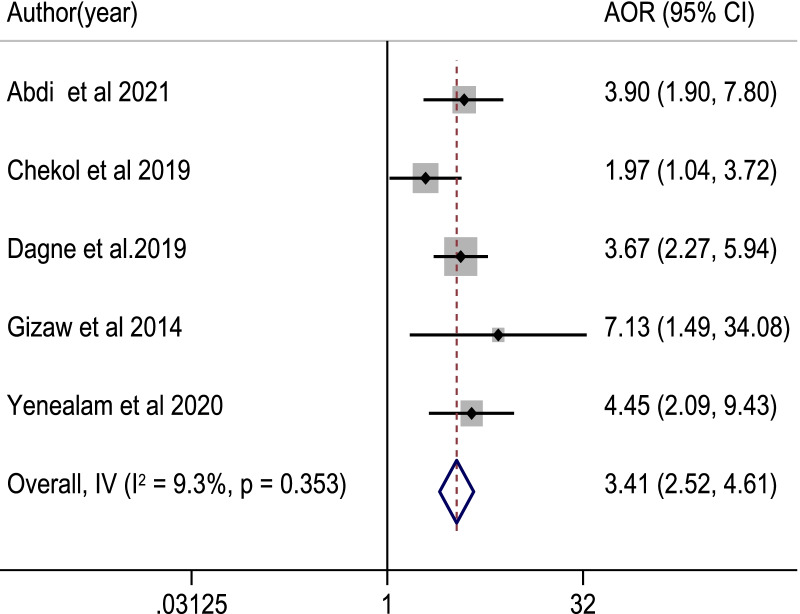
The pooled adjusted odds ratio of the association between positive attitude of food handlers and food hygiene practice in Ethiopia

**Fig. 7:**
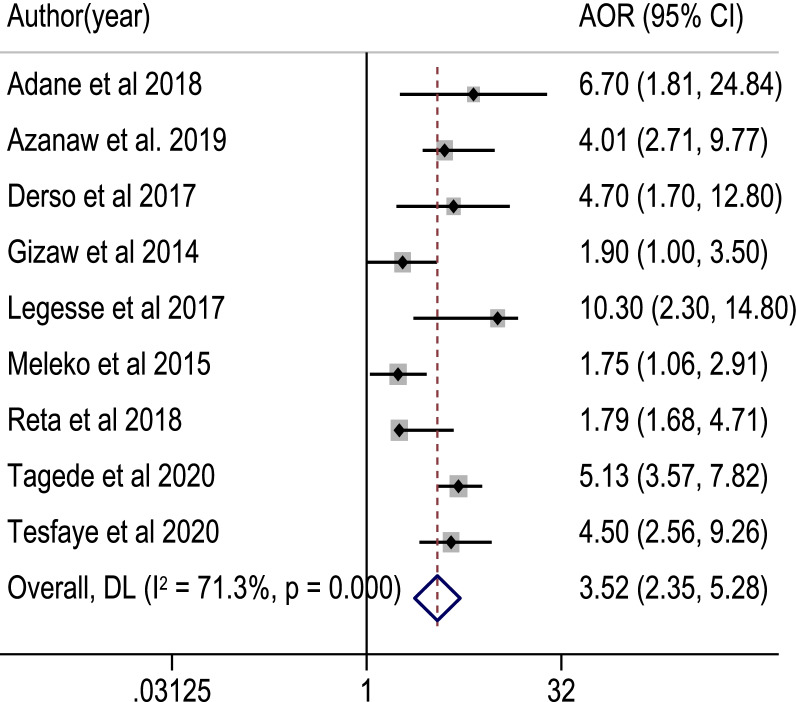
The pooled adjusted odds ratio of the association between training of food handlers and food hygiene practice in Ethiopia

**Fig. 8:**
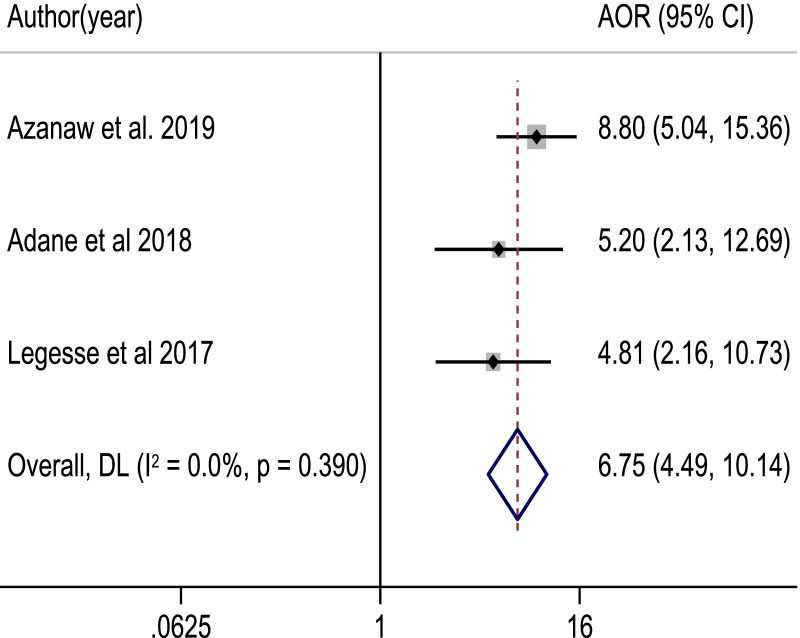
The pooled adjusted odds ratio of the association between routine medical checkup of food handlers and food hygiene practice in Ethiopia

**Fig. 9:**
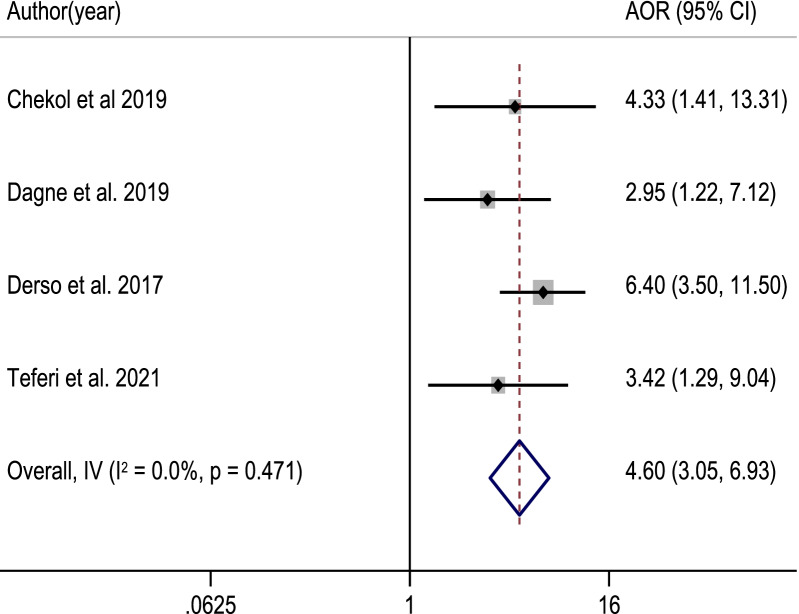
The pooled adjusted odds ratio of the association between educational status of food handlers and food hygiene practice in Ethiopia

Using nine critical studies [34, 38, 41, 43, 51–55], we looked at the relationship between knowledge of food handlers on main food hygiene components and food hygiene practices in this meta-analysis. Accordingly, food handlers with good knowledge of food hygiene were nearly two times more likely to practice good food hygiene than their counterparts (POR: 1.98, 95% CI: 1.26, 3.11). The test statistics revealed high heterogeneity among the included studies (*I*^2^ = 82.4% and *p* < 0.001). As a result, the association was determined using a random effect model (Fig. 5). Similarly, five studies [37, 41, 43, 52, 53] examined the association between a positive attitude and good food hygiene practice. Food handlers with a positive attitude were 3.4 times more likely to have good food hygiene practices than those with a negative attitude (POR: 3.41, 95% CI: 2.52, 4.61). A fixed-effect model was applied, because there was lower heterogeneity among the studies (*I*^2^ = 9.3% and *p* = 0.353) (Fig. 6). Nine studies [34, 38, 42, 44, 46, 50, 51, 53, 54] were used to observe the relationship between training on food hygiene components and good food hygiene practice. The likelihoods of good food hygiene practice were 3.5 times higher among trained food handlers than those who had not received training (POR: 3.52, 95% CI: 2.35, 5.28). The random-effect model was used, because there was moderate heterogeneity among the included studies (*I*^2^ = 71.3% and *p* < 0.001) (Fig. 7). On the other hand, three studies [38, 42, 46] were used to determine the association between good food hygiene practice and receiving routine medical checkups. As a result, food handlers who had routine medical checkups were 6.75 times more likely to have good food hygiene practice than their counterparts (POR: 6.75, 95% CI: 4.49, 10.14). There was lower heterogeneity in the included studies (*I*^2^ = 0.0% and *p* = 0.390), a fixed-effect model was used (Fig. 8). Four studies [37, 43, 44, 57] were considered to indicate the association between good food hygiene practice and educational status food handlers. The odds of having good food hygiene practice were higher among food handlers who had formal education in relation to those who had no formal education (POR = 4.60, 95% CI: 3.05, 6.93). There was no heterogeneity in the included studies (*I*^2^ = 0.0% and *p* = 0.471), a fixed-effect model was used (Fig. 9).

## Discussion

Food contamination and outbreaks of food borne diseases are largely determined by food handlers' understanding and food hygiene practice, particularly in LMICs, such as Ethiopia, where food hygiene regulations are negligent [58, 59]. Food safety standards are the foundation for limiting disease transmission from food handlers to consumers [20]. The objective of this systematic review and meta-analysis was to determine the pooled proportion of good food hygiene practices and its determinants in Ethiopia. In this review, the overall proportion of food handlers who had good food hygiene practices was 50.2%. Although improper food handling techniques are the root cause of the vast majority of foodborne diseases [23, 60], barely half of the Ethiopian food handlers adopt good food hygiene. This finding may be due to inadequate training of food handlers, poor infrastructure, and the regulatory team's irregular/weak supervision of food establishments. Another explanation for this finding is that food handlers may not have consistently followed all food safety/hygiene guidelines, such as personal hygiene, utensil cleaning, and sanitization, adequate cooking, avoiding cross-contamination, storing foods at appropriate temperatures, and avoiding food from potentially unsafe sources [61, 62].

Variation between studies resulted in high heterogeneity in our study. As a result, we performed a sub-group analysis using a region, in which the Amhara region had the highest proportion of good food hygiene practice, while the SNNPR had the lowest proportion. In comparison to research conducted in other regions, most of the studies included in this review were from the Amhara region, and different types or levels of food establishments may explain the regional discrepancies. Another reason for the disparity could be related to differences in food handlers' experience, training, and behavioral characteristics. As a result of our findings, it might be necessary to encourage the desired degree of good food hygiene practice in all Ethiopian regions.

A subgroup analysis was also done on the study area and sampling method. As a result, studies conducted only in universities revealed a larger proportion of good food hygiene practices than studies conducted in cities, which revealed a lower proportion. This difference might be due to food handlers in universities closely followed by health professionals to practice all recommended food hygiene components, and uniform customer served in university. Compared to census and systematic sampling, studies with systematic random sampling had a higher proportion of good food hygiene practices. These variations could be attributed to disparities in distinctive properties of food handler obligations, training, and sample methods distinctive properties.

The second objective of this review was to determine whether factors were associated with good food hygiene practices among Ethiopian food handlers. As a result, food handlers' knowledge, attitude, training regarding food hygiene components, and regular medical checkups were substantially associated with good food hygiene practices. Food handlers who had routine medical checkups had a higher chance of having good food hygiene practices than those who had occasional medical checkups. This variation could be because food handlers are aware of food hygiene components during regular medical checkups. On the other hand, the relationship between being trained on food hygiene components and good food hygiene practice was considered. Food handlers who had received training were more likely to follow good food hygiene practices than those who had not trained. Therefore, providing food handlers with food hygiene training is crucial for enhancing practical skills and guaranteeing good food hygiene practices.

Furthermore, food safety training is the most extensively utilized technique to improve good food hygiene practices [63–65]. Studies conducted in Bangladesh [66], Saudi Arabia [67], Korea [68], and Brazil [62] support that food handlers who had received training were more likely to follow good food hygiene practices. Similarly, food handlers with good knowledge were more likely to conduct good food hygiene practices than those with poor knowledge. This variation could be explained as adequate knowledge is important and putting that knowledge into practice is even more imperative [69]. This finding of good knowledge levels among food handlers to have good hygiene practices was supported by research from Ethiopia [70], Brazil [24], and in the British [71].

Food handlers who had a positive attitude towards food hygiene components had a higher likelihood of good food hygiene practice than those who had a negative attitude. Therefore, it signifies that food handlers with a positive attitude toward food hygiene practices exhibit positive behaviors [9, 72].

## Implication of the finding

Foodborne disease continues to be a major public health concern around the world. Despite significant progress in strengthening food safety systems, foodborne infections affect one-third of the population of affluent countries each year, and the problem is expected to be far more common in poorer countries. To preserve consumer confidence in the food safety system and to create a sound regulatory foundation for domestic and international food trade that supports economic development, it is critical to assess the degree of food hygiene practice. Improving modifiable risk variables such as food handler training, attitude, and awareness of food handlers have a role in decreasing foodborne illness. Our study find out the important factors of the good food hygiene practice, which will aids in the implementation of feasible interventions to promote food handler compliance with food hygiene components.

## Limitations

There are certain limits to this study. First, all the included studies were cross-sectional in design, making it difficult to establish cause–effect relationships. Second, the proportion of good food hygiene among food handlers was determined in all studies based on self-reporting, which may overestimate food handlers' actual practice. Third, there is no gold standard definition used for ‘good food hygienic practices,’ and thus, it slightly varies between studies. Finally, only articles written in English were taken into account. Thus, the future researchers should focus on observation studies with strong design, such as cohort and interventional studies.

## Conclusions

In this review, only half of the food handlers in Ethiopia had good food hygiene practices, and there were regional variation in good food hygiene practices among food handlers. The study concluded that food handlers with routine medical checkups, training, education, and a favorable attitude toward food hygiene components were all associated factors with good food hygienic practices. This research can generate a framework for food handlers, policymakers, and other stakeholders to implement evidence-based interventions. More emphasis should be placed on aggregating excellent food hygiene practices by enhancing food handlers' knowledge, attitude, and on-the-job and off-the-job training, as this is a critical method to avoid poor food hygiene practices.

## Supplementary Information

**Additional file 1. **PRISMA checklist.

**Additional file 2.** Risk of bias assessment of included studies.

**Additional file 3. **Summary finding table (grade).

## Data Availability

The manuscript contains all pertinent information.

## References

[1] Grace D. Food safety in developing countries: research gaps and opportunities.

[2] World Health Organization. National systems to support drinking-water: sanitation and hygiene: global status report 2019: UN-Water global analysis and assessment of sanitation and drinking-water: GLAAS 2019 report.

[3] Biran A, Curtis V, Gautam OP, Greenland K, Islam MS, Schmidt WP, Sijbesma C, Sumpter C, Torondel B (2012). Background paper on measuring WASH and food hygiene practices–definition of goals to be tackled post-2015 by the Joint Monitoring Programme. London Sch Hyg Trop Med.

[4] Angelo K, Nisler A, Hall A, Brown L, Gould L (2017). Epidemiology of restaurant-associated foodborne disease outbreaks, United States, 1998–2013. Epidemiol Infect.

[5] Havelaar AH, Kirk MD, Torgerson PR, Gibb HJ, Hald T, Lake RJ, Praet N, Bellinger DC, De Silva NR, Gargouri N, Speybroeck N (2015). World Health Organization global estimates and regional comparisons of the burden of foodborne disease in 2010. PLoS Med.

[6] Grace D (2015). Food safety in low and middle-income countries. Int J Environ Res Public Health.

[7] Mudey AB, Kesharwani N, Mudey GA, Goyal RC, Dawale AK, Wagh VV (2010). Health status and personal hygiene among food handlers working at food establishment around a rural teaching hospital in Wardha District of Maharashtra, India. Global J Health Sci.

[8] Callejón RM, Rodríguez-Naranjo MI, Ubeda C, Hornedo-Ortega R, Garcia-Parrilla MC, Troncoso AM (2015). Reported foodborne outbreaks due to fresh produce in the United States and European Union: trends and causes. Foodborne Pathog Dis.

[9] Hanson LA, Zahn EA, Wild SR, Döpfer D, Scott J, Stein C (2012). Estimating global mortality from potentially foodborne diseases: an analysis using vital registration data. Popul Health Metrics.

[10] Thelwell-Reid M (2014). Food safety knowledge and self-reported practices of food handlers in Jamaica.

[11] Zaglool D, Khodari Y, Othman R, Farooq M (2011). Prevalence of intestinal parasites and bacteria among food handlers in a tertiary care hospital. Niger Med J.

[12] Ayana Z, Yohannis M, Abera Z (2015). Food-borne bacterial diseases in Ethiopia. Acad J Nutr.

[13] Saeed HA, Hamid HH (2010). Bacteriological and parasitological assessment of food handlers in the Omdurman area of Sudan. J Microbiol Immunol Infect.

[14] Kassani A, Shaterian M, Sharifirad G, Menatid R, Abbastabar H, Ebrahimipour M (2015). The prevalence of some intestinal parasites in food-handlers of Asian and African countries: a meta-analysis. Arch Hyg Sci.

[15] Wadilo F, Solomon F, Arota A, Abraham Y (2016). Intestinal parasitic infection and associated factors among food handlers in South Ethiopia: a case of Wolaita Sodo town. J Pharm Altern Med.

[16] Gezehegn D, Abay M, Tetemke D, Zelalem H, Teklay H, Baraki Z (2017). Prevalence and factors associated with intestinal parasites among food handlers of food and drinking establishments in Aksum Town, Northern Ethiopia. BMC Public Health.

[17] Tefera T, Mebrie G (2014). Prevalence and predictors of intestinal parasites among food handlers in Yebu Town, southwest Ethiopia. PLoS ONE.

[18] Wegayehu T, Tsalla T, Seifu B, Teklu T (2013). Prevalence of intestinal parasitic infections among highland and lowland dwellers in Gamo area South Ethiopia. BMC Public Health.

[19] Chaib F, Lawe-Davies O. WHO’s First-Ever Global Estimates of Foodborne Diseases Find Children under 5 Account for Almost One-Third of Death.

[20] Motarjemi Y, Lelieveld H (2013). Food safety management: a practical guide for the food industry.

[21] Takalkar A, Madhekar N, Kumavat A, Bhayya S (2010). Prevalence of intestinal parasitic infections amongst food handlers in hotels and restaurants in Solapur city. Indian J Public Health.

[22] Mendedo EK, Berhane Y, Haile BT (2017). Factors associated with sanitary conditions of food and drinking establishments in Addis Ababa, Ethiopia: a cross-sectional study. Pan Afr Med J.

[23] Trigunarso SI (2020). Sanitation hygiene and food handling behavior with the number of germs on snack foods in the school environment/Hygiene Sanitasi dan Perilaku Penjamah Makanan dengan Angka Kuman pada Makanan Jajanan di Lingkungan Sekolah. J Kesehatan.

[24] Zanin LM, da Cunha DT, de Rosso VV, Capriles VD, Stedefeldt E (2017). Knowledge, attitudes and practices of food handlers in food safety: an integrative review. Food Res Int.

[25] Kaferstein FK, Abdussalam M. Food safety in the 21st century/F. Käferstein and M. Abdussalam. Food safety in the 21st century/F Käferstein and M Abdussalam1999.

[26] Lee HK, Abdul Halim H, Thong KL, Chai LC (2017). Assessment of food safety knowledge, attitude, self-reported practices, and microbiological hand hygiene of food handlers. Int J Environ Res Public Health.

[27] Soares LS, Almeida RC, Cerqueira ES, Carvalho JS, Nunes IL (2012). Knowledge, attitudes and practices in food safety and the presence of coagulase-positive staphylococci on hands of food handlers in the schools of Camaçari Brazil. Food Control.

[28] Fasanmi O, Makinde G, Popoola M, Fasina O, Matere J, Ogundare S (2018). Potential risk factors associated with carcass contamination in slaughterhouse operations and hygiene in Oyo state. Nigeria. Int J Livestock Prod.

[29] Evans H, Madden P, Douglas C, Adak G, O'Brien S, Djuretic T (1998). General outbreaks of infectious intestinal disease in England and Wales, 1995 and 1996. Commun Dis Public Health.

[30] Smith SI, Agomo CO, Bamidele M, Opere BO, Aboaba OO (2010). Survey of food handlers in bukas (a type of local restaurant) in Lagos, Nigeria about typhoid fever. Health.

[31] Al-Shabib NA, Mosilhey SH, Husain FM (2016). A cross-sectional study on food safety knowledge, attitude, and practices of male food handlers employed in restaurants of King Saud University, Saudi Arabia. Food Control.

[32] Wambui J, Karuri E, Lamuka P, Matofari J (2017). Good hygiene practices among meat handlers in small and medium enterprise slaughterhouses in Kenya. Food Control.

[33] Yeshanew S, Tadege M, Abamecha A (2021). Prevalence and associated factors of intestinal parasitic infections among food handlers in Mettu Town, Southwest Ethiopia. J Trop Med.

[34] Tesfaye A, Tegene Y (2020). Assessment of food hygiene and safety practices among street food vendors and its associated factors in urban areas of Shashemane, West Arsi Zone, Oromia Ethiopia, 2019. J Biomed Res Environ Sci.

[35] Kuti KA, Nur RA, Donka GM, Kerbo AA, Roba AE (2020). Predictors of intestinal parasitic infection among food handlers working in Madda Walabu University, Ethiopia: a cross-sectional study. Interdiscip Perspect Infect Dis.

[36] Kibret M, Abera B (2012). The sanitary conditions of food service establishments and food safety knowledge and practices of food handlers in Bahir Dar town. Ethiop J Health Sci.

[37] Chekol FA, Melak MF, Belew AK, Zeleke EG (2019). Food handling practice and associated factors among food handlers in public food establishments, Northwest Ethiopia. BMC Res Notes.

[38] Azanaw J, Gebrehiwot M, Dagne H (2019). Factors associated with food safety practices among food handlers: a facility-based cross-sectional study. BMC Res Notes.

[39] Stern C, Lizarondo L, Carrier J, Godfrey C, Rieger K, Salmond S (2020). Methodological guidance for the conduct of mixed methods systematic reviews. JBI Evid Synth.

[40] Higgins J, Altman D, Gotzsche P, Juni P, Moher D, Oxman A (2011). The Cochrane Collaboration's tool for assessing the risk of bias in randomized trials. BMJ.

[41] Abdi AM, Amano A, Abrahim A, Getahun M, Ababor S, Kumie A (2020). Food hygiene practices and associated factors among food handlers working in food establishments in the Bole Sub City, Addis Ababa. Ethiopia. Risk Manag Healthc Policy.

[42] Adane M, Teka B, Gismu Y, Halefom G, Ademe M (2018). Food hygiene and safety measures among food handlers in street food shops and food establishments of Dessie town, Ethiopia: a community-based cross-sectional study. PLoS ONE.

[43] Dagne H, Raju RP, Andualem Z, Hagos T, Addis K. Food safety practice and its associated factors among mothers in Debarq town, northwest Ethiopia: community-based cross-sectional study. BioMed Research International. 2019;2019. 10.1155/2019/1549131 PMC6582849 31275961

[44] Derso T, Tariku A, Ambaw F, Alemenhew M, Biks GA, Nega A (2017). Socio-demographic factors and availability of piped fountains affect food hygiene practice of food handlers in Bahir Dar Town, Northwest Ethiopia: a cross-sectional study. BMC Res Notes.

[45] Lalit I, Brkti G, Dejen Y. Magnitude of hygienic practices and its associated factors of food handlers working in selected food and drinking establishments in Mekelle town, northern Ethiopia. Int Food Res J. 2015;22(6):2650–6.

[46] Legesse D, Tilahun M, Agedew E, Haftu D (2017). Food handling practices and associated factors among food handlers in arba Minch town public food establishments in Gamo Gofa Zone, Southern Ethiopia. Epidemiology (Sunnyvale).

[47] Lema K, Abuhay N, Kindie W, Dagne H, Guadu T (2020). Food hygiene practice and its determinants among food handlers at University of Gondar, Northwest Ethiopia, 2019. Int J Gen Med.

[48] Mardu F, Negash H, Legese H, Berhe B, Tesfay K, Haileslasie H (2020). Assessment of knowledge, practice, and status of food handlers toward Salmonella, Shigella, and intestinal parasites: a cross-sectional study in Tigrai prison centers, Ethiopia. PLoS ONE.

[49] Mekasha T, Neela S, Kumela D (2016). Food safety knowledge, practice and attitude of food handlers in traditional hotels of Jimma Town, Southern Ethiopia. Annals Food Science and Technology.

[50] Meleko A, Henok A, Tefera W, Lamaro T (2015). Assessment of the sanitary conditions of catering establishments and food safety knowledge and practices of food handlers in Addis Ababa University Students’ Cafeteria. Science.

[51] Reta MA, Lemma MT, Gemeda AA, Lemlem GA. Food handling practice and associated factors among food handlers working in food establishments in Woldia town, Northeast Ethiopia. 10.11604/pamj.2021.40.128.19757 PMC8641629 34909096

[52] Yenealem DG, Yallew WW, Abdulmajid S (2020). Food safety practice and associated factors among meat handlers in gondar town: a cross-sectional study. J Environ Public Health.

[53] Gizaw Z, Gebrehiwot M, Teka Z (2014). Food safety practice and associated factors of food handlers working in substandard food establishments in Gondar Town Northwest Ethiopia. Int J Food Sci Nutr Diet.

[54] Alemayehu T, Aderaw Z, Giza M, Diress G (2021). Food safety knowledge, handling practices and associated factors among food handlers working in food establishments in Debre Markos Town, Northwest Ethiopia, 2020: institution-based cross-sectional study. Risk Manag Healthc Policy.

[55] Tessema AG, Gelaye KA, Chercos DH (2014). Factors affecting food handling Practices among food handlers of Dangila town food and drink establishments North West, Ethiopia. BMC Public Health.

[56] Abe S, Arero G (2021). Food handler’s safety practices and related factors in the public food establishments in Batu Town, Central Oromia, Ethiopia. Health.

[57] Teferi SC, Sebsibe I, Adibaru B (2021). Food safety practices and associated factors among food handlers of Fiche Town, North Shewa Zone, Ethiopia. J Environ Public Health.

[58] Dagnew M, Tiruneh M, Moges F, Tekeste Z (2012). Survey of nasal carriage of Staphylococcus aureus and intestinal parasites among food handlers working at Gondar University, Northwest Ethiopia. BMC Public Health.

[59] Odeyemi OA (2016). Public health implications of microbial food safety and foodborne diseases in developing countries. Food Nutr Res.

[60] Soon J, Singh H, Baines R (2011). Foodborne diseases in malaysia: a review. Food Control.

[61] Lee HK, Abdul Halim H, Thong KL, Chai LC (2017). Assessment of food safety knowledge, attitude, self-reported practices, and microbiological hand hygiene of food handlers. Int J Environ Res Public Health.

[62] Medeiros L, Hillers V, Kendall P, Mason A (2001). Evaluation of food safety education for consumers. J Nutr Educ.

[63] Medeiros CO, Cavalli SB, Salay E, Proença RPC (2011). Assessment of the methodological strategies adopted by food safety training programs for food service workers: a systematic review. Food Control.

[64] da Cunha DT, Stedefeldt E, de Rosso VV (2014). The role of theoretical food safety training on Brazilian food handlers' knowledge, attitude, and practice. Food Control.

[65] Egan M, Raats M, Grubb S, Eves A, Lumbers M, Dean M (2007). A review of food safety and food hygiene training studies in the commercial sector. Food Control.

[66] Uddin MJ, Koehlmoos TL, Ashraf A, Khan A, Saha NC, Hossain M (2009). Health needs and health-care-seeking behavior of street-dwellers in Dhaka, Bangladesh. Health Policy Plan.

[67] Amer OH, Ashankyty IM, Haouas NAS (2016). Prevalence of intestinal parasite infections among patients in local public hospitals of Hail, Northwestern Saudi Arabia. Asian Pac J Trop Med.

[68] Park S-H, Kwak T-K, Chang H-J (2010). Evaluation of the food safety training for food handlers in restaurant operations. Nurs Res Pract.

[69] Acikel CH, Ogur R, Yaren H, Gocgeldi E, Ucar M, Kir T (2008). The hygiene training of food handlers at a teaching hospital. Food Control.

[70] Teferi SC (2020). A review on food hygiene knowledge, practice and food safety in Ethiopia. Sci J Food Sci Nutr.

[71] McIntyre L, Vallaster L, Wilcott L, Henderson SB, Kosatsky T (2013). Evaluation of food safety knowledge, attitudes and self-reported handwashing practices in trained and untrained food handlers in British Columbia, Canada. Food Control.

[72] Lestantyo D, Husodo AH, Iravati S, Shaluhiyah Z (2017). Safe food handling knowledge, attitude and practice of food handlers in the hospital kitchen. Int J Public Health Sci.

