# Supplementary material for: Food hygiene practices and determinants among food handlers in Ethiopia: a systematic review and meta-analysis
Source: Trop Med Health. 2022 May 19;50:34. doi: 10.1186/s41182-022-00423-6 (PMC9118835; doi:10.1186/s41182-022-00423-6)
Supplement: Supplementary file 2 — Additional file 2. Risk of bias assessment of included studies. [file 41182_2022_423_MOESM2_ESM.docx]

**Additional file 1, list of excluded references and reasons for exclusion.**

| **References** | **Reasons** |
| --- | --- |
| 1. Alemnew B, Gedefaw G, Diress G, Bizuneh AD. Prevalence and factors associated with intestinal parasitic infections among food handlers working at higher public University student's cafeterias and public food establishments in Ethiopia: a systematic review and meta-analysis. BMC Infect Dis. 2020;20(1):156. | 1 |
| 1. Alemu AS, Baraki AG, Alemayehu M, Yenit MK. The prevalence of intestinal parasite infection and associated factors among food handlers in eating and drinking establishments in Chagni Town, Northwest Ethiopia. BMC research notes. 2019;12(1):1-6. | 1 |
| 1. Amare A, Worku T, Ashagirie B, Adugna M, Getaneh A, Dagnew M. Bacteriological profile, antimicrobial susceptibility patterns of the isolates among street vended foods and hygienic practice of vendors in Gondar town, Northwest Ethiopia: a cross sectional study. BMC microbiology. 2019;19(1):1-9. | 1 |
| 1. Asires A, Wubie M, Reta A. Prevalence and associated factors of intestinal parasitic infections among food handlers at prison, east and west Gojjam, Ethiopia. Advances in medicine. 2019;2019. | 1 |
| 1. Bafa TA, Sherif EM, Hantalo AH, Woldeamanuel GG. Magnitude of enteropathogens and associated factors among apparently healthy food handlers at Wolkite University Student's Cafeteria, Southern Ethiopia. BMC research notes. 2019;12(1):567-. | 1 |
| 1. Berhanu L, Mereta ST, Gume B, Kassa T, Berihun G, Dadi LS, et al. Effect of Microbial Quality of Washing Water on Hand Hygiene Status of Food Handlers in Jimma Town: Implication for Food Hygiene and Safety. Journal of multidisciplinary healthcare. 2021;14:1129-34. | 1 |
| 1. Berhe AA, Aregay AD, Abreha AA, Aregay AB, Gebretsadik AW, Negash DZ, et al. Knowledge, Attitude, and Practices on Water, Sanitation, and Hygiene among Rural Residents in Tigray Region, Northern Ethiopia. Journal of environmental and public health. 2020;2020:5460168-9. | 1 |
| 1. Diriba K, Awulachew E, Ashuro Z. Prevalence and antimicrobial resistance pattern of Salmonella, Shigella, and intestinal parasites and associated factor among food handlers in Dilla University student cafeteria, Dilla, Ethiopia. International journal of microbiology. 2020;2020. | 1 |
| 1. Gemeda T, Asayehu T, Abdisa M, Fekadu H. Assessment of knowledge, attitude and practices of food handlers in Nekemte Referral Hospital, Wollega, Ethiopia. J Nutr Health Food Eng. 2018;8(1):00262. | 1 |
| 1. Gezehegn D, Abay M, Tetemke D, Zelalem H, Teklay H, Baraki Z, et al. Prevalence and factors associated with intestinal parasites among food handlers of food and drinking establishments in Aksum Town, Northern Ethiopia. BMC public health. 2017;17(1):1-9. | 1 |
| 1. Girmay AM, Gari SR, Mengistie Alemu B, Evans MR, Gebremariam AG. Determinants of sanitation and hygiene status among food establishments in Addis Ababa, Ethiopia. Environmental health insights. 2020;14:1178630220915689. | 1 |
| 1. Gutema FD, Agga GE, Abdi RD, Jufare A, Duchateau L, De Zutter L, et al. Assessment of Hygienic Practices in Beef Cattle Slaughterhouses and Retail Shops in Bishoftu, Ethiopia: Implications for Public Health. International journal of environmental research and public health. 2021;18(5):2729. | 1 |
| 1. Haileselassie M, Taddele H, Adhana K, Kalayou S. Food safety knowledge and practices of abattoir and butchery shops and the microbial profile of meat in Mekelle City, Ethiopia. Asian Pacific Journal of Tropical Biomedicine. 2013;3(5):407-12. | 1 |
| 1. Hajare ST, Gobena RK, Chauhan NM, Erniso F. Prevalence of Intestinal Parasite Infections and Their Associated Factors among Food Handlers Working in Selected Catering Establishments from Bule Hora, Ethiopia. BioMed Research International. 2021;2021. | 1 |
| 1. Kebede E, Seid A, Akele S. Prevalence and associated risk factors of intestinal parasitic infections among asymptomatic food handlers in Wollo University student’s cafeteria, Northeastern Ethiopia. BMC research notes. 2019;12(1):1-6. | 1 |
| 1. Kumie A, Mezene A, Amsalu A, Tizazu A, Bikila B. The sanitary condition of food and drink establisment in Awash-Sebat Kilo town, Afar Region, Ethiopia. Ethiopian Journal of Health Development. 2006;20(3). | 1 |
| 1. Kumma WP, Meskele W, Admasie A. Prevalence of intestinal parasitic infections and associated factors among food handlers in Wolaita Sodo University students caterings, Wolaita Sodo, Southern Ethiopia: A cross-sectional study. Frontiers in Public Health. 2019;7:140. | 1 |
| 1. Mama M, Alemu G. Prevalence and factors associated with intestinal parasitic infections among food handlers of Southern Ethiopia: cross sectional study. BMC public health. 2015;16(1):1-7. | 1 |
| 1. Mendedo EK, Berhane Y, Haile BT. Factors associated with sanitary conditions of food and drinking establishments in Addis Ababa, Ethiopia: cross-sectional study. Pan Afr Med J. 2017;28:237. | 1 |
| 1. Mengeda TJ, Gesese SA, Geleta FT, Geleta BD, Gemeda DE. Food Safety Awareness and Practices of Food Handlers in Cafes and Restaurants of Ambo, Guder and Ginchi Towns of West Shoa Zone, Oromia National Regional State, Ethiopia. Health. 2020;3:2.5. | 1 |
| 1. Mulat M, Desta T, Birri D. Food safety knowledge and practice among food handlers in Yeka Subcity, Addis Ababa, Ethiopia. International Journal of Infectious Diseases. 2020;101:418. | 1 |
| 1. Neme K, Hailu B, Belachew T. assess sanitary condition and food handling practices of restaurants in Jimma Town, Ethiopia: implication for food born infection and food intoxication. Food Sci Qual Manag. 2017;60:2225-0557. | 1 |
| 1. Nigusse D, Kumie A. Food hygiene practices and prevalence of intestinal parasites among food handlers working in Mekelle university student’s cafeteria, Mekelle. Global Advanced Research Journal of Social Science. 2012;1(4):65-71. | 1 |
| 1. Tefera T, Mebrie G. Prevalence and predictors of intestinal parasites among food handlers in Yebu Town, southwest Ethiopia. PloS one. 2014;9(10):e110621. | 1 |
| 1. Tegegne HA, Berhanu A, Getachew Y, Serda B, Nölkes D, Tilahun S, et al. Microbiological safety and hygienic quality of camel meat at abattoir and retail houses in Jigjiga city, Ethiopia. J Infect Dev Ctries. 2019;13(3):188-94. | 1 |
| 1. Tegegne HA, Phyo HWW. Food safety knowledge, attitude and practices of meat handler in abattoir and retail meat shops of Jigjiga Town, Ethiopia. J Prev Med Hyg. 2017;58(4):E320-e7. | 1 |
| 1. Mardu F, Negash H, Legese H, Berhe B, Tesfay K, Haileslasie H, et al. Assessment of knowledge, practice, and status of food handlers toward Salmonella, Shigella, and intestinal parasites: A cross-sectional study in Tigrai prison centers, Ethiopia. PloS one. 2020;15(11):e0241145. | 1 |
| 1. Zegeye A. A note on the influence of heat treatment, salting and smoking on the acceptability of camel meat products. Meat Sci. 1999;53(4):217-9. | 1 |
| 1. Tilahun W, Endebu T, Abera T. Health Status of Food Handlers and Associated Factors at Hotels and Restaurants in Adama Town, Ethiopia.PLoS One. 2021;16(4):e0250020. | 1 |
| 1. Aragaw M, Tafese T, Beyene Z, Hailemariam Z, Azaze A, Luce R, et al. Shigellosis outbreak at Addis Ababa University: March-April 2010. Ethiopian medical journal. 2011;49(4):341-8. | 1 |

**Reasons for exclusion: 1. the outcome of interests was not reported**
