# Supplementary material for: Food hygiene practices and determinants among food handlers in Ethiopia: a systematic review and meta-analysis
Source: Trop Med Health. 2022 May 19;50:34. doi: 10.1186/s41182-022-00423-6 (PMC9118835; doi:10.1186/s41182-022-00423-6)
Supplement: Supplementary file 3 — Additional file 3. Summary finding table (grade). [file 41182_2022_423_MOESM3_ESM.docx]

| **Summary finding table on selected study reported determinants for good food hygiene practice among food handlers** | | | | | | |
| --- | --- | --- | --- | --- | --- | --- |
| **Patient or population:** good food hygiene practice among food handlers  **Settings:** Ethiopia **Intervention:** Determinants | | | | | | |
| **Outcomes** | **Illustrative comparative risks* (95% CI)** | | **Relative effect (95% CI)** | **No of Participants (studies)** | **Quality of the evidence (GRADE)** | **Comments** |
|  | Assumed risk | Corresponding risk |  |  |  |  |
|  | **Control** | **Determinants** |  |  |  |  |
| **Training[Adane et al 2021]** | **Study population** | | **OR 6.7**  (1.8 to 24.8) | 135 (1 study) | ⊕⊕⊝⊝ **low**^1,2^ |  |
|  | See comment | See comment |  |  |  |  |
|  | **Moderate** | |  |  |  |  |
|  |  |  |  |  |  |  |
| **Training[Derso et al 2017]** | **Study population** | | **OR 4.7**  (1.7 to 12.8) | 417 (1 study) | ⊕⊕⊝⊝ **low** |  |
|  | See comment | See comment |  |  |  |  |
|  | **Moderate** | |  |  |  |  |
|  |  |  |  |  |  |  |
| **Training [Gizaw et al 2014]** | **Study population** | | **OR 1.9**  (1.01 to 3.5) | 403 (1 study) | ⊕⊕⊝⊝ **low** |  |
|  | See comment | See comment |  |  |  |  |
|  | **Moderate** | |  |  |  |  |
|  |  |  |  |  |  |  |
| **Training[Meleko et al 2015]** | **Study population** | | **OR 1.8**  (1.1 to 2.9) | 302 (1 study) | ⊕⊕⊝⊝ **low** |  |
|  | See comment | See comment |  |  |  |  |
|  | **Moderate** | |  |  |  |  |
|  |  |  |  |  |  |  |
| **Training[Reta et al 2018]** | **Study population** | | **OR 1.8**  (1.7 to 4.7) | 288 (1 study) | ⊕⊕⊝⊝ **low** |  |
|  | See comment | See comment |  |  |  |  |
|  | **Moderate** | |  |  |  |  |
|  |  |  |  |  |  |  |
| **Training[Alemayehu et al 2020]** | **Study population** | | **OR 2.1**  (1.3 to 3.2) | 408 (1 study) | ⊕⊕⊕⊝ **moderate**^3^ |  |
|  | See comment | See comment |  |  |  |  |
|  | **Moderate** | |  |  |  |  |
|  |  |  |  |  |  |  |
| **Training[Tesfaye et al 2020]** | **Study population** | | **OR 4.5**  (2.6 to 9.3) | 120 (1 study) | ⊕⊕⊝⊝ **low** |  |
|  | See comment | See comment |  |  |  |  |
|  | **Moderate** | |  |  |  |  |
|  |  |  |  |  |  |  |
| **Attitude[Chekol et al 2019]** | **Study population** | | **OR 1.9**  (1.01 to 3.7) | 416 (1 study) | ⊕⊕⊝⊝ **low** |  |
|  | See comment | See comment |  |  |  |  |
|  | **Moderate** | |  |  |  |  |
|  |  |  |  |  |  |  |
| **Attitude[Dagne et al.2019]** | **Study population** | | **OR 3.7**  (2.3 to 5.9) | 423 (1 study) | ⊕⊕⊝⊝ **low** |  |
|  | See comment | See comment |  |  |  |  |
|  | **Moderate** | |  |  |  |  |
|  |  |  |  |  |  |  |
| **Attitude[Yenealam et al 2020]** | **Study population** | | **OR 4.5**  (2.1 to 9.4) | 214 (1 study) | ⊕⊕⊝⊝ **low** |  |
|  | See comment | See comment |  |  |  |  |
|  | **Moderate** | |  |  |  |  |
|  |  |  |  |  |  |  |
| **Knowledge[Abdi et al 2021]** | **Study population** | | **OR 3.3**  (1.9 to 5.8) | 394 (1 study) | ⊕⊕⊕⊝ **moderate**^3^ |  |
|  | See comment | See comment |  |  |  |  |
|  | **Moderate** | |  |  |  |  |
|  |  |  |  |  |  |  |
| **Knowledge[Azanaw et al. 2019]** | **Study population** | | **OR 2.9**  (1.4 to 4.1) | 384 (1 study) | ⊕⊕⊕⊝ **moderate**^3^ |  |
|  | See comment | See comment |  |  |  |  |
|  | **Moderate** | |  |  |  |  |
|  |  |  |  |  |  |  |
| **Knowledge[Tessema et al 2020]** | **Study population** | | **OR 1.7**  (1.1 to 2.7) | 406 (1 study) | ⊕⊕⊝⊝ **low** |  |
|  | See comment | See comment |  |  |  |  |
|  | **Moderate** | |  |  |  |  |
|  |  |  |  |  |  |  |
| **Education[Teferi et al. 2021]** | **Study population** | | **OR 3.4**  (1.3 to 9.0) | 422 (1 study) | ⊕⊕⊕⊝ **moderate**^3^ |  |
|  | See comment | See comment |  |  |  |  |
|  | **Moderate** | |  |  |  |  |
|  |  |  |  |  |  |  |
| **Medical check up[Legesse et al 2017]** | **Study population** | | **OR 4.8**  (2.2 to 10.7) | 383 (1 study) | ⊕⊕⊕⊝ **moderate**^3^ |  |
|  | See comment | See comment |  |  |  |  |
|  | **Moderate** | |  |  |  |  |
|  |  |  |  |  |  |  |
| *The basis for the **assumed risk** (e.g. the median control group risk across studies) is provided in footnotes. The **corresponding risk** (and its 95% confidence interval) is based on the assumed risk in the comparison group and the **relative effect** of the intervention (and its 95% CI).  **CI:** Confidence interval; **OR:** Odds ratio; | | | | | | |
| GRADE Working Group grades of evidence **High quality:** Further research is very unlikely to change our confidence in the estimate of effect.  **Moderate quality:** Further research is likely to have an important impact on our confidence in the estimate of effect and may change the estimate. **Low quality:** Further research is very likely to have an important impact on our confidence in the estimate of effect and is likely to change the estimate. **Very low quality:** We are very uncertain about the estimate. | | | | | | |
| ^1^ No explanation was provided ^2^ Odds ratio is greater than 5 ^3^ Odds ratio is greater than 2 | | | | | | |
